# Multiple Variables at the Leukocyte Cell Surface Impact Fc γ Receptor-Dependent Mechanisms

**DOI:** 10.3389/fimmu.2019.00223

**Published:** 2019-02-14

**Authors:** Kashyap R. Patel, Jacob T. Roberts, Adam W. Barb

**Affiliations:** Roy J. Carver Department of Biochemistry, Biophysics, and Molecular Biology, Iowa State University, Ames, IA, United States

**Keywords:** antibody, IgG, N-glycosylation, post-translation modification, ADCC—antibody dependent cellular cytotoxicity, immune complex, ADCP—antibody dependent cellular phagocytosis

## Abstract

Fc γ receptors (FcγR) expressed on the surface of human leukocytes bind clusters of immunoglobulin G (IgG) to induce a variety of responses. Many therapeutic antibodies and vaccine-elicited antibodies prevent or treat infectious diseases, cancers and autoimmune disorders by binding FcγRs, thus there is a need to fully define the variables that impact antibody-induced mechanisms to properly evaluate candidate therapies and design new intervention strategies. A multitude of factors influence the IgG-FcγR interaction; one well-described factor is the differential affinity of the six distinct FcγRs for the four human IgG subclasses. However, there are several other recently described factors that may prove more relevant for disease treatment. This review covers recent reports of several aspects found at the leukocyte membrane or outside the cell that contribute to the cell-based response to antibody-coated targets. One major focus is recent reports covering post-translational modification of the FcγRs, including asparagine-linked glycosylation. This review also covers the organization of FcγRs at the cell surface, and properties of the immune complex. Recent technical advances provide high-resolution measurements of these often-overlooked variables in leukocyte function and immune system activation.

## Introduction: The Importance of Modulating the Fc-FcγR Interaction

**Figure 1:**
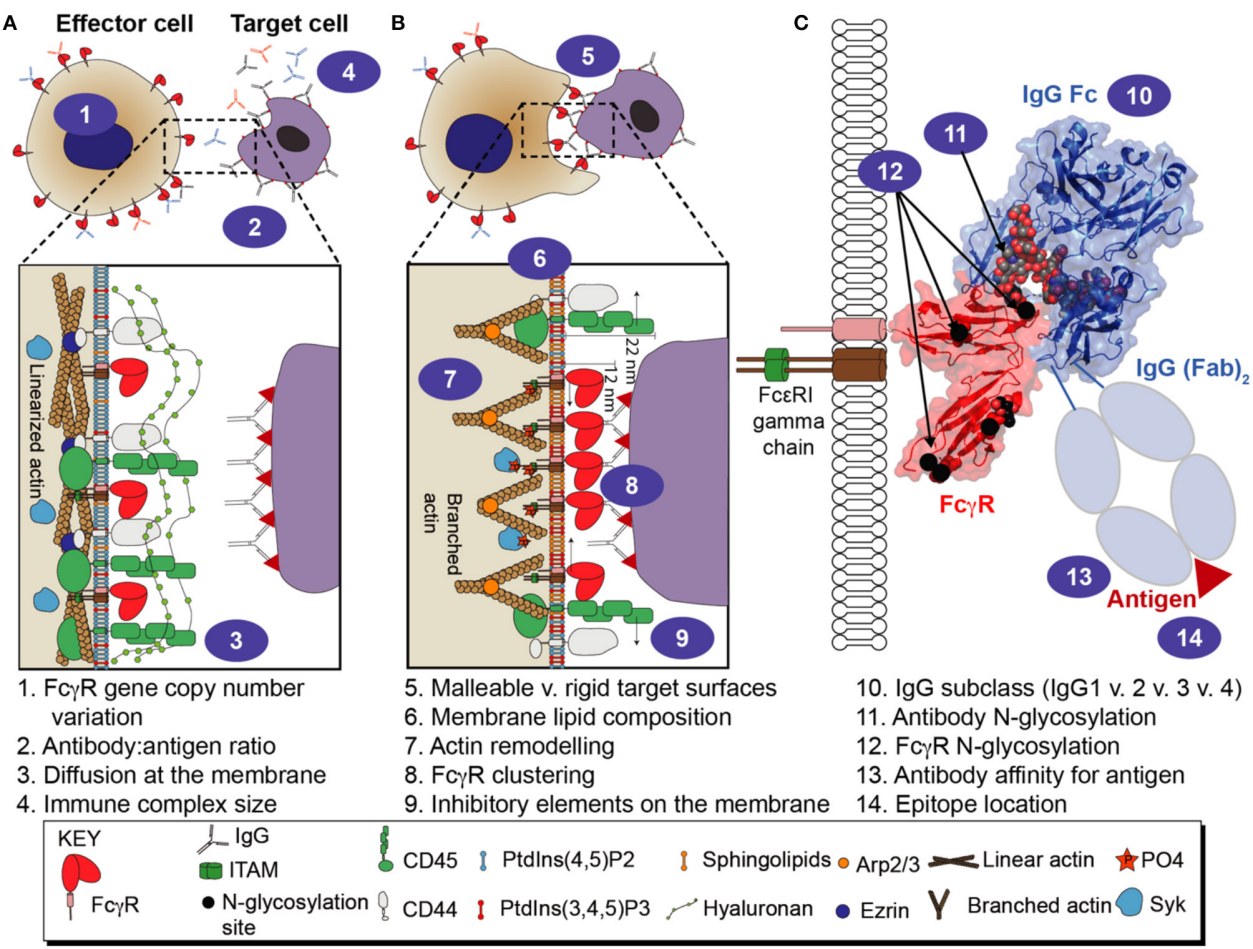
Multiple variables affect FcγR-mediated immune function. **(A)** cellular variables influencing FcγR activity that are present before the effector cell engages a target cell. **(B)** cellular variables influencing FcγR-mediated activity while the effector cell is engaged with a target cell. **(C)** molecular variables associated with the FcγRs, antibody, and antigen.

**Figure 2:**
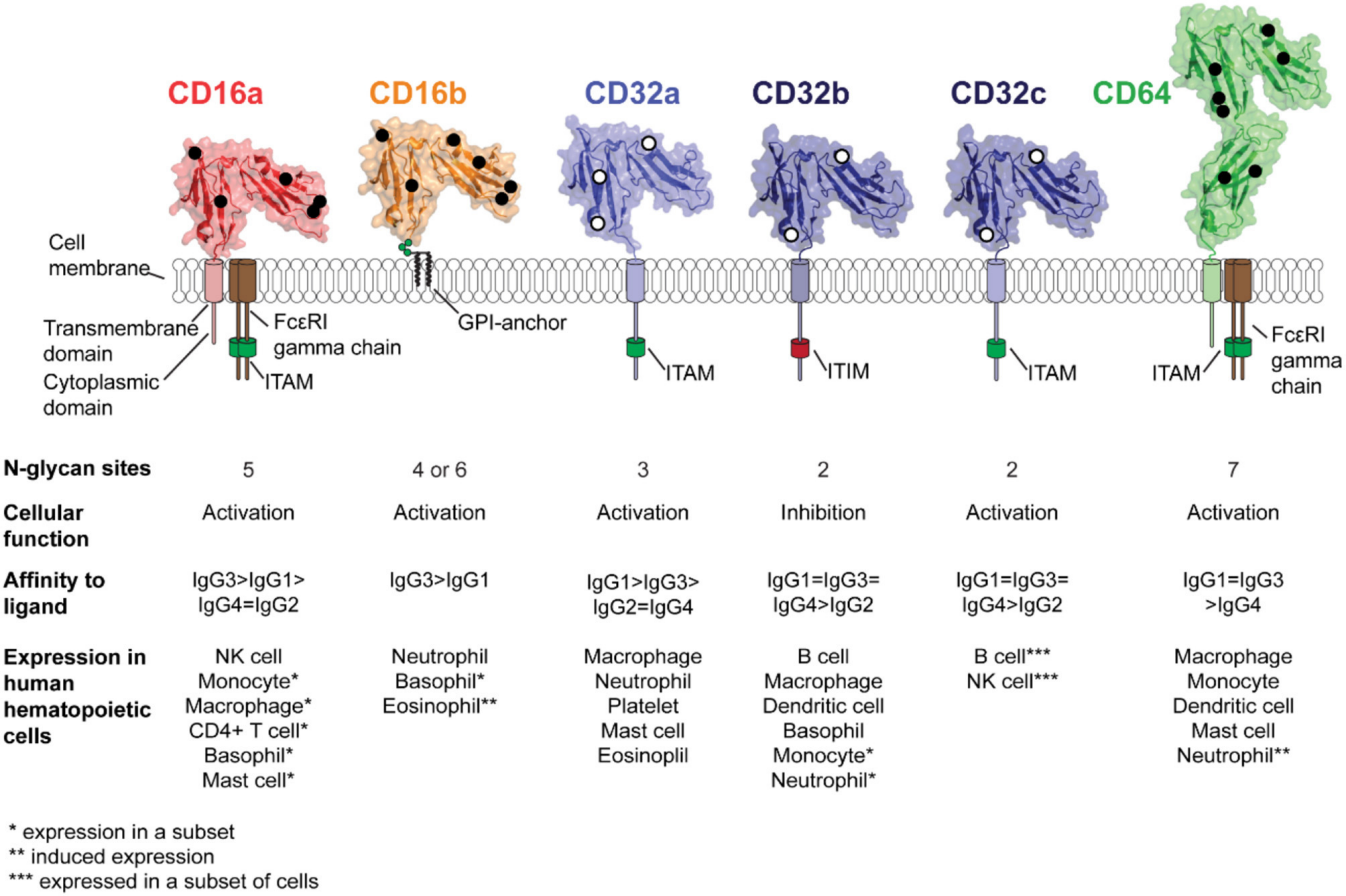
Structures and properties of the human Fc γ receptors. Five receptors are expressed in the majority of the population with CD32c expressed only in a small subset. The ribbon diagrams show the structures of the extracellular antibody-binding domains as determined by x-ray crystallography; overlayed black or white circles indicate the sites of N-glycosylation.

Immunoglobulin G (IgG) is the most thoroughly studied and well characterized molecule of the humoral immune response. IgG activates the immune system through cell-bound Fc γ Receptors (FcγRs; Figure 1). The IgG fragment antigen-binding (Fab) domains confer specificity and affinity toward an antigen while the distinct hinge and fragment crystallizable (Fc) domain of the four IgG subclasses (IgG1-4) provide the structural basis for specificity and affinity to bind FcγRs (1). The six structurally distinct members of the classical human FcγRs (FcγRI or CD64, FcγRIIa/CD32a, FcγRIIb/CD32b, FcγRIIc/CD32c, FcγRIIIa/CD16a, and FcγRIIIb/CD16b) are expressed on leukocytes of both the myeloid and lymphoid lineage (Figure 2). This group of proteins can be divided into two types: activating receptors (CD64, CD32a, CD32c, CD16a, and CD16b) that lead to cell activation through immunoreceptor tyrosine-based activation motifs (ITAM) on cytosolic tails or on co-receptor molecules, and an inhibitory receptor (CD32b) that signals through immunoreceptor tyrosine-based inhibitory motifs (ITIM) (2–4). Only CD32s contain ITAM or ITIM domains, and the other receptors must associate with an ITAM-containing adaptor protein (FcεRI γ chain or CD3 ζ chain) (3, 5) (Figure 2). In either situation, the ratio of activating to inhibiting signals determines the outcome of an immune response (6).

Receptor clustering is essential for FcγR signaling. Circulating IgG coats an antigen to form an oligomeric complex, positioning the Fc portions of the IgG molecules away from the target surface and exposed to interact with FcγRs. The antibody-coated target is also referred to as an immune complex. The multiple IgG molecules of the immune complex provide an opportunity for multivalent interactions with FcγR-expressing leukocytes and must compete with non-complexed serum antibodies occupying the FcγRs that will, in turn, cluster FcγRs on the cell surface (7, 8). Depending on the receptors engaged, the clustering of the extracellular domains triggers phosphorylation of tyrosine in the ITAMs or ITIMs, which subsequently recruits signaling molecules that promote a cellular response (9). The types of FcγR-mediated effector cell responses are diverse and include, but are not limited to, antibody-dependent cellular cytotoxicity (ADCC), antibody-dependent cellular phagocytosis (ADCP), release of cytokines and antigen uptake for presentation (10–14). FcγRs are critical for maintaining immune system homeostasis as well as preventing pathogenic infections and they play a major role in inflammatory diseases and autoimmune disorders (9, 13, 15–17). The combination of distinct antagonistic and synergistic factors contribute to a considerable functional diversity within this group of antibody receptors. Here we will discuss multiple factors which influence the antibody:FcγR interaction and modify the immune response (Figure 1).

## Receptor Presentation at the Cell Surface

FcγRs are predominately expressed on cells originating from hematopoietic progenitor stem cells including dendritic cells, neutrophils, basophils, eosinophils, macrophages, monocytes, mast cells, NK cells, B cells, a subset of T cells, and platelets as well as non-hematopoietic cell types such as syncytiotrophoblasts at various levels (18–20). FcγR expression varies depending on cell lineage; not surprisingly gene copy number is also implicated in disease. These factors can greatly influence the dynamic ability of the immune system to respond to the diverse repertoire of foreign invaders. Thus, variable surface expression by different immune cell types influences how the immune system responds to a foreign invader. This section will cover the cellular expression of FcγRs and immune modulation of expression through downregulation and induction.

Five activating FcγRs are expressed in humans (Figure 2). The highest affinity, CD64, is expressed on monocytes, dendritic cells and macrophages (11), mast cells (21), and neutrophils following IFN-γ exposure (22, 23). The low affinity CD32a is expressed on mast cells, neutrophils, macrophages, eosinophils, and platelets (24). CD32c is expressed by 7–15% of individuals on NK cells and B cells and results from a gene mutation (4). The high/moderate affinity CD16a is expressed predominantly on NK cells, a subset of monocytes, mast cells, basophils, macrophages and is inducible in CD4+ T-cells (25, 26). The low/moderate affinity CD16b is found only in humans and expressed predominantly on neutrophils (27), a subset of basophils (28) and has inducible expression on eosinophils (29, 30). CD32b is the sole inhibitory receptor and is expressed on basophils, B cells, macrophages, dendritic cells, a subset of monocytes and neutrophils (24). Interestingly, CD32b is also expressed in non-hematopoeitic cells, including the endothelium of various organs (31).

### Variability in Receptor Amount

Gene duplications in individuals lead to copy number variation (CNV) of some FcγRs in the population. Surprisingly, only CD16a, CD16b, and CD32c of the FcγRs exhibited CNV in a sample population of 600 subjects (32). CNVs have been correlated to autoimmune disorders as well as variations in surface expression levels. CNV of CD16b is correlated to surface expression on neutrophils and implicated in SLE susceptibility (33, 34), as well as other autoimmune disorders (35, 36). Furthermore, CD16a CNV appears to be functionally significant since increased surface expression positively correlated with increasing CD16a gene number (ranging from one to three copies) (32, 35). A CD16a indel has been shown to increase surface expression as well (37).

FcγR amount at the cell surface varies by cell type and receptor identity (Figure 2). On neutrophils, there are an estimated 100,000–300,000 surface exposed CD16b molecules and 10,000–40,000 CD32a molecules (38, 39). The predominant monocyte subtype at roughly 80% of the pool, “classical” monocytes, does not express CD16a. “Non-classical” monocytes express CD16a at a level of roughly 10,000 CD16a molecules per cell but upon differentiation into macrophages express 40,000 CD16a molecules per cell while CD32 remained the same at ~10,000 molecules per cell (40). Another study found macrophages express 5–10 fold higher CD64, CD32a, and CD32b while CD16a expression was comparable to non-classical monocytes. M2c macrophages expressed overall higher levels of FcγRs than M1 macrophages with the following order of expression: CD32a, CD32b > CD64 > CD16a (41). A high number of CD16a molecules are expressed on CD16+ NK cells (100,000-250,000) (42).

Expression levels also vary based on the cell status. Following activation, innate immune cells can induce expression of FcγRs (23, 25, 29, 30, 35). There is also evidence of receptor downregulation upon activation. Downregulation mechanisms include both decreases in expression as well as shedding FcγR from the cell surface following metalloproteinase cleavage. CD32a is shed from Langerhans cells and also expressed as a soluble form (43). CD32b is shed upon activation of B-cells (44). CD16a and CD16b are likewise shed upon activation of NK cells and neutrophils at a known cleavage site by the metalloprotease ADAM17 (45–48). Intriguingly, sCD16b is relatively abundant in serum (~5 nM) (49) and levels vary based on the immune state of the individual (50). Surprisingly, CD64 is the only human FcγR in which a soluble, serum-borne form has not been reported. This may be explained by the presence of a third extracellular CD64 domain in place of the cleavage site found in CD32s and CD16s (Figure 2).

Soluble FcγR forms modulate immune responses. Soluble CD16b binds myeloid cells, NK cells, subsets of T cells, B cells, and monocytes through complement receptor 3 (CR3 or Mac-1 or αM β2, comprised of CD11b/CD18) and complement receptor 4 (CR4 or αx β2, comprised of CD11c/CD18). These interactions cause the release of IL6 and IL8 by monocytes and indicate a potential role for soluble CD16b in inflammation (51). Shedding of CD16a from NK cells allows disengagement of the immune synapse from the target cell and the subsequent ability to kill again. One study demonstrated that repeated engagement by CD16a depleted perforin, however, shedding of CD16a allowed perforin replenishment upon subsequent activation by another activating receptor, Natural killer group 2 member D (NKG2D), which recognizes ligands not normally expressed on healthy tissue (52). Thus, it appears that the act of shedding of CD16 can allow disengagement of the foreign particle which would be crucial for the immune cell's survival and preservation of potential future cytolytic activity. Though shed receptors are proinflammatory and recruit immune cells as discussed above, a complete picture of the mechanisms of regulating surface expression upon immune activation is not currently available.

### Receptor Clustering at the Membrane Is Required for Effector Function

The correct presentation of FcγRs on the cell membrane is essential for proper immune cell function. ADCC can destroy virally infected cells and cancer cells, and is thus a target for monoclonal antibody (mAb) therapies (53). ADCP is also an important mechanism in mAb therapy targeting malignant cells (14). ADCC and ADCP are dependent on the ability of low to moderate affinity FcγRs to cluster on fluid plasma membranes for activation to occur (54) (Figure 1). Equally important is the regulation of these receptors when no activation signal is present.

Proper activation of FcγRs following Fc engagement by macrophages requires clustering of FcγRs and the displacement of inhibitory receptors. In one study utilizing murine RAW 264.7 cells, segregation of CD45, a phosphatase responsible for dephosphorylating ITAMs, is dependent on antigen distance from the target membrane (55) (Figure 1). It appears that if the antibody is >10 nm from the target surface, there is a substantially impaired ADCP response. This phenomenon is due to the location of the epitope; epitopes closer to the surface exclude the inhibitory CD45 molecule (which stands ~22 nm tall vs. FcγR-IgG complex = 11.5 nm) from the immune synapse. Interestingly, a follow-up study that focused on FDA-approved mAbs found the targets were small surface proteins (< 10 nm in height) suggesting there may be a requirement for mAb epitopes to be located close to the surface for therapeutic efficacy. CD45 was also excluded from the immune synapse in activated human T cells (56). Another study concerning inhibitory module segregation on human macrophages demonstrates CD64, but not CD32a, and inhibitory signal regulating protein α (SIRPα), in conjunction with CD47 (a receptor that inhibits macrophage phagocytosis), are clustered on quiescent cells but upon activation segregate in a process regulated by spleen tyrosine kinase (SYK)-dependent actin cytoskeleton reorganization (57). Recently, FcγR diffusion has been shown to be inhibited by the CD44 transmembrane protein which is immobilized by linearized actin filaments via ezrin/radixen/moesin (ERM) and binds hyaluronan in the glycocalyx (58). This study used primary human macrophages as well as murine cell lines and murine models, utilizing single particle tracking found CD44 and hyaluronan decreased the diffusion rate of FcγRs, while also sterically blocking the binding of FcγRs to immune complexes (Figure 1).

Receptor clustering overwhelms constitutive inhibition as described previously, allowing phosphorylation of the ITAM. ITAMs are phosphorylated via SYK, Src family kinase (SFKs) or ζ-chain-associated protein kinase 70 (ZAP-70) for downstream activation of phosphoinositide-3-kinase (PI3K), NF-κB, extracellular signal regulated kinase (ERK), phosphatidyl inositol 4-phosphate 5-kinase γ (PIP5Kγ), GTPases and other SRC-family kinases (53, 54, 59, 60). Along with FcγR clustering, actin polymerization and depolymerization is equally important for phagocytosis in RAW 264.7 macrophages by creating lammellipodium/pseudopods. These protrusions are controlled by Rac GTPase and lipid composition (54, 59) (Figure 1). Clustering has also been observed on the plasma membrane of murine derived macrophages using total internal reflection microscopy (TIRF) of a lipid bilayer supporting IgG (61). The FcγR microcluster appears on the macrophage pseudopod edge and is subsequently transported to a synapse-like structure thereby recruiting SYK and production of PtdIns(3–5)P3 coordinated with lamellar actin polymerization. Another study on quiescent human macrophages found lateral diffusion of FcγRs is regulated by tonic activity of SYK causing actin cytoskeleton organization to increase the likelihood of FcγRs to be pre-clustered upon finding a pathogen (62).This study further described differential FcγR mobility upon activation. FcγRs at the periphery of the actin-rich pseudopod were more mobile than those already immobilized by binding of IgG-rich regions. The authors explained that this mobility difference is controlled by SYK-mediated regulation of the actin-cytoskeleton which would increase the likelihood of FcγRs to engage more IgG molecules at the leading edge of the lamellipodium/pseudopod and not waste time diffusing into already IgG-dependent, FcγR-immobilized, actin-rich rich regions of plasma membrane. Mobility of FcγRs was described earlier to be decreased at the trailing end of polarized macrophages by CD44 that was bound to linear actin and connected to hyaluronan (58). It was also found in this study that on the leading edge of polarized macrophages, the side that encounters opsonized material, Arp2/3-driven actin branching predominates, initiated by phosphotidlyinosotide (3–5)-trisphosphate production, and increased FcγR mobility allowing for more efficient clustering at the immune synapse. When Arp2/3-driven actin branching predominates, it was found CD44 is more mobile allowing greater FcγR mobility (Figure 1).

In the human NK92 cell line, transduced to express CD16a, a study showed β2 integrins mediate the dynamics of FcγR receptor microclusters in a protein-tyrosine kinase 2 (Pyk2)-dependent manner, controlling the rate of target cell destruction by ADCC (63). β2 integrins bind ICAM-1 on the target cell allowing adhesion and signal transduction through Pyk2 for actin remodeling and the subsequent enhancement of FcγR mobility. Furthermore, sites of granule release are surrounded by clusters of CD16a and release points are devoid of actin. Human NK cell lytic granules also converge at the surface in a dynein and integrin-signal dependent manner which aids spatial targeting of the weaponized molecules to limit off-target damage (64). Surprisingly, CD16a is essential for ADCC of human CD16+ monocytes and upon CD16a engagement, β2 integrins are activated along with TNFα secretion thereby indicating that non-classical monocytes (CD16+) are the sole monocyte class capable of ADCC (65).

During the early stages of phagocytosis by RAW 264.7 cells, direct contact between FcγRs and IgG is increased by greater IgG density on particles, and increased IgG density results in an increased level of early signals. However, late stage signals are “all or nothing,” not concentration dependent, and regulated by PI3K concentration in the phagocytic membranes (66). In this study, low IgG density decreased the amount of opsonized particles but not the rate of phagosome formation and low IgG density particles that did result in phagocytosis recruited the same amounts of late stage signaling molecules (PIP3, Protein kinase C ε type, p85 subunit) and actin. Overall it appears that FcγRs control the initial binding process essential for scanning the foreign particle and initial activation by binding IgG and later stages of commitment to destruction of the particle are controlled by both IgG density and membrane lipid composition.

On murine and human macrophages, receptor clustering upon activation is consistent with a change in the heterogeneity of the membrane lipid composition to a highly ordered phagosomal membrane that is heavily enriched in sphingolipids and ceramide but lacking cholesterol (67) (Figure 1). The authors state that lipid remodeling mediates F-actin remodeling and the biophysical characteristics of the phagosomal membrane are essential for phagocytosis. On human B cells, a polymorphism of the inhibitory receptor CD32b (Ile232Thr) located in the middle of the transmembrane domain, is described to decrease inhibitory function (68). This mutation was shown to result in aberrant localization to a sphingolipid and cholesterol rich region in contrast to the Ile232 wild-type. Aberrant localization is not surprising considering the introduction of a polar residue into the transmembrane domain (69). Furthermore, the ability of CD32b to inhibit B cell receptor (BCR)-mediated PIP3 production, AKT, phospholipase C-γ-2 (PLCγ2) activation and calcium mobilization was impaired in cells expressing the CD32b Thr232 allotype as compared to Ile232. The authors indicate the FcγR locus was associated with SLE and this polymorphism may promote disease. Thus, it appears lipid composition is important for FcγR-mediated mechanisms.

The unique construction of CD16b indicates the potential for a different activation mechanism for neutrophils. Neutrophils predominantly express CD16b with 10-fold less CD32a. CD32a signal transduction is well described and thought to be the canonical FcγR signal transduction via phosphorylation of ITAMs and subsequent SYK recruitment (70). However, CD16b contains a GPI anchor and does not have a polypeptide transmembrane domain nor is it known to associate with a signaling coreceptor, therefore, it is unclear how CD16b promotes signaling in neutrophils (Figure 2). CD16b plays a role in the initial binding of immune complexes in concert with β2 integrins (71). Currently there are conflicting studies suggesting that CD16b can transduce a signal on its own (70, 72, 73), or it transduces a signal by acting with CD32a (74). A recent study found CD16b cross-linking increased IL-10 and TNFα expression, phosphorylated SHP-2 in a lipid-raft mediated manner and inhibited apoptosis in neutrophils. Lipid composition certainly may be an important part of CD16b signal transduction in mechanisms similar to those discussed previously for macrophage phagocytosis and CD32b on B-cells, however the role of lipids in neutrophil activation is not understood (75–81). Interestingly proteinase 3 (PR3), CD16b, cytochrome b558, and NADPH oxidase co-immunoprecipitate on lipid rafts and PR3 and CD16b colocalize in confocal imaging suggesting these may interact in a lipid raft (75). Other findings suggest CD16b signals in conjunction with CR3 via lectin-like interactions (82), leading to neutrophil respiratory bursts (72). The function of GPI-linked CD16b remains undefined despite the high abundance of CD16b in the body and critical roles in mAb therapies (83).

### The Type of FcγR Membrane Anchor Impacts Activation

There are clear differences between the signaling and antibody-binding affinity of soluble and membrane-anchored FcγR forms. However, less is known about the effects of the specific FcγR membrane anchors on affinity and cell activation. All FcγRs are localized to the membrane by a transmembrane polypeptide moiety or a glycosylphosphatidylinositol (GPI) moiety (CD16b only) (Figure 2). A micropipette adhesion assay demonstrated CD16a attached to microspheres via a GPI anchor bound roughly 5-fold tighter to IgG1-coated red blood cells (RBCs) than CD16a tethered by a transmembrane domain (84, 85). Interestingly, it also appears IgG1-coated spheres treated with phosphoinositide phospholipase C (PIPLC) to remove the diacylglycerol moiety bound to GPI-linked CD16a with 12-fold less affinity. These authors observed a 60-fold decrease when the GPI-anchor was completely removed. A CD16b-GPI construct showed 2-fold decrease of affinity upon PIPLC treatment and an 11-fold decrease following removal of the GPI-anchor. The authors hypothesized that enhancement of binding affinity associated with the GPI anchor may be due to an allosteric effect on CD16, changing the structure to bind IgG more effectively; such an allosteric mechanism was observed with other GPI-anchored proteins (80). Further studies will be required to fully elucidate how the GPI-anchor affects CD16b and how specific aspects of the membrane anchor confers distinct properties *in vivo*.

## Post-Translational Modification of the Antibody and Receptor

**Figure 3:**
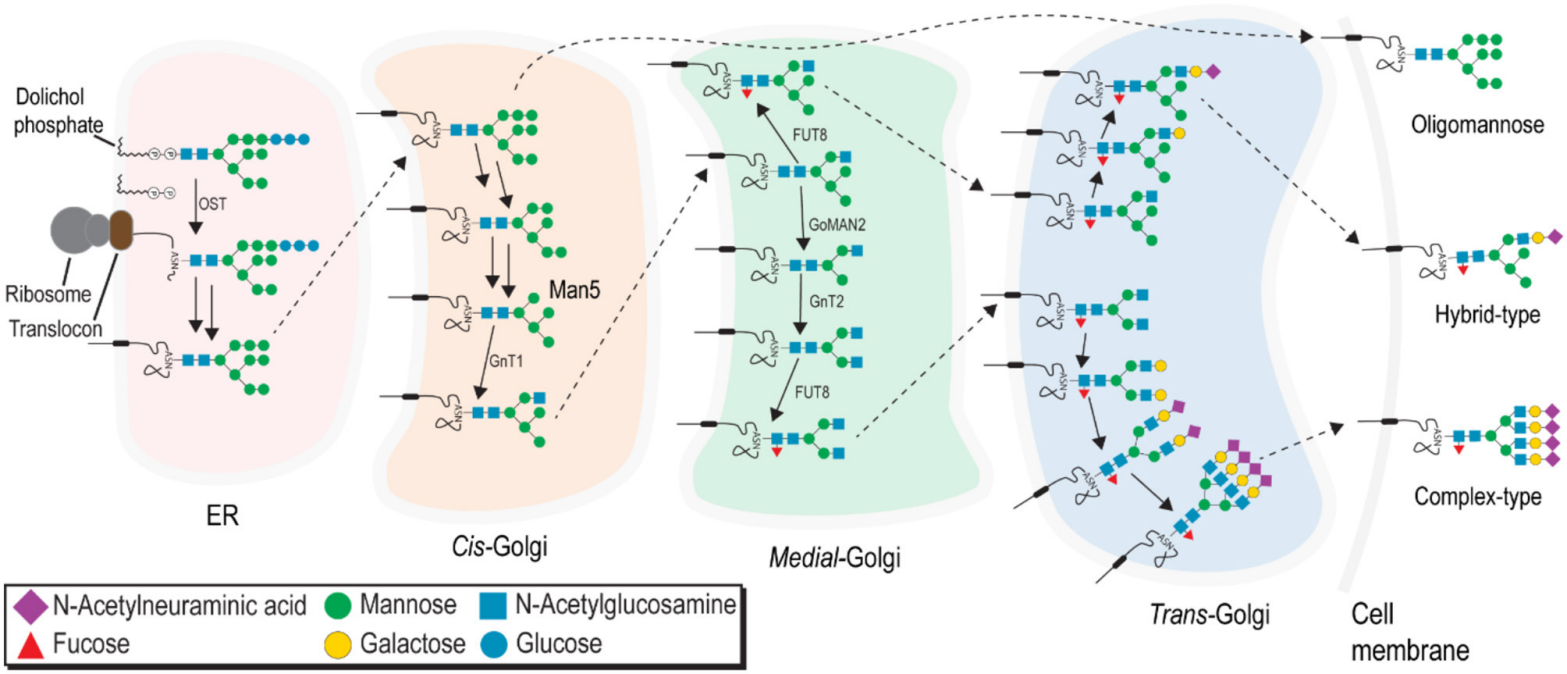
N-glycans are processed in the ER and Golgi to produce three major classes: minimally processed oligomannose-type N-glycans, fully processed complex-type N-glycans, and hybrid-type N-glycans with an intermediate level of processing.

Asparagine-linked (N-) glycosylation is one of the most common protein modifications performed by the eukaryotic cell and is a substantial modification of all FcγRs [Figure 2; for a thorough review of N-glycan processing, see (86)].It is important to note, however, the resulting glycans processed in the ER and Golgi can be grouped into three distinct forms: (1) minimally-processed oligomannose type N-glycans, (2) intermediate processed hybrid-type N-glycans with processing on one of the two core mannose branches, and (3) highly-processed complex-type N-glycans with extensively modified branches (Figure 3).

Several variables introduce a significant degree of heterogeneity into the N-glycan present at any single site on a glycoprotein, ranging from substrate availability, protein anchor type, to accessibility of N-glycan site, potentially creating a vast diversity of protein forms and functions (87–90). This heterogeneity also renders glycoproteins challenging targets for *in vitro* studies to characterize structure. Minimally-processed hybrid and oligomannose type N-glycans are not expected at the cell surface because these forms harbor terminal mannose residues that may bind to the mannose receptor and elicit an immune response (91, 92). Though many previous glycomics studies report high levels of oligomannose N-glycans recovered from primary cells, the abundance of these under-processed forms is likely due to cell lysis and recovery of unprocessed glycans from the ER. If under-processed forms are present on the cell surface, these must be protected from binding to the mannose receptor. Therefore, highly processed complex-type N-glycans are expected as the predominant species at the cell surface.

The functional impact of N-glycosylation at the conserved asparagine 297 residues in IgG1 is well established. IgG1 glycosylation at Asn-297 is essential for the IgG-FcγR interaction (93). The N-glycosylation profile of serum IgG changes due to multiple factors, including age, gender, infection, pregnancy, and disease (94–97). The variation in IgG1 Fc glycoforms is known to change antibody affinity toward the FcγRs (98), and this fact has also been leveraged to develop glycoengineered mAbs and anti-inflammatory glycoforms of intravenous immunoglobulin (IVIG) (99, 100). The wealth of knowledge regarding IgG glycoforms is due in large part to protein abundance and ease of obtaining samples. However, little is known about the glycosylation of FcγRs on immune cells.

FcγRs are heavily glycosylated molecules, containing two to seven N-glycans (Figure 2). The extent of FcγR modification was evident as early as 1988 as certain FcγRs from native tissue migrated much slower in SDS-PAGE gels than expected based on the polypeptide mass alone. Furthermore, the migration rate increased after treatment to specifically remove N-glycans (101, 102). There is a prominent gap in knowledge about the impact of FcγR N-glycosylation on immune function largely due to limited studies of the native FcγRs purified from primary leukocytes. However, it is known that CD16a expressed by NK cells had a distinct N-glycosylation profile when compared CD16a expressed by cultured monocytes, though this determination was made using lectin binding (103) and surface CD16a on the NK cell and monocyte displayed differential antibody-binding affinity that was attributed to differences in cell-specific CD16a N-glycosylation (104).

Even though native glycoforms of all FcγRs are not known, the effect of N-glycosylation on binding affinity has been well characterized *in vitro* using protein expressed with mammalian cells. Aglycosylated, recombinant, soluble (s)FcγRs bind IgG1 Fc at different affinities than glycosylated forms, thus the IgG-FcγR interaction is sensitive to receptor N-glycosylation (105–107). Recent studies reported substantial differences in affinity for sFcγRs expressed in recombinant systems (106, 108–110), N-glycosylation profiles of NK cell CD16a and soluble CD16b from serum revealed surprising heterogeneity and substantial differences from recombinantly-expressed protein (109, 111).

### Specific CD16a Glycoforms Bind Antibody With High Affinity Comparable to CD64

The analysis of N-glycan composition from FcγRs provides a characteristic profile of a protein (112). Glycomics analysis of CD16a on circulating NK cells from three healthy donors revealed a surprising abundance of under-processed forms (~45% hybrid and oligomannose-type N-glycans). CD16a is N-glycosylated at five sites (Figure 2). The remainder of the N-glycans were primarily complex type, biantennary N-glycan structures with a high degree of sialylation (78%) and fucosylation (89%) (109). The under-processed forms do not likely originate from unprocessed CD16a in the ER because all of the observed hybrid forms were sialylated, a modification that occurs in the late Golgi compartments (113) (Figure 3). Moreover, the presence of oligomannose type N-glycans on CD16a from almost all recombinant sources suggests that restricted processing is a conserved feature (108–110). N-glycans at Asn38 and Asn74 were not observed using this glycomics approach to study NK cell CD16a; perhaps these large glycans ionize too poorly to be observed in a derivatized form, but robust ionization of the peptide provides measurable signals for CD16b N38 and N74 glycopeptides (111).

Recombinant expression has thus far failed to generate CD16 with glycan profiles matching those measured for CD16a or CD16b from primary cells. CD16a is the most heavily studied FcγR due to its role in ADCC and the associated therapeutic applications. Glycomics characterization of soluble extracellular domain of CD16a (sCD16a) from HEK293, NS0, and CHO cell lines showed stark differences when compared to CD16a from NK cells, including a high abundance (over 90% compared to 55% in NK cells) of biantennary and triantennary complex type N-glycans with low levels of sialylation (108–110). Moreover, each recombinant system has the potential to synthesize unique N-glycan structures that are not commonly found on native human proteins, such as LacDiNAc (GlcNAc-GalNAc) from HEK293 cells, α-Gal epitopes (αGal-βGal-βGlcNAc), terminal N-acetylglycolylneuraminic acid in NS0 cells and only α-2,3 linked sialic acids in CHO cells (106, 114). These terminal modifications can potentially alter the binding affinity to IgG in an unexpected and undesirable manner.

Differences between native and recombinant CD16a processing render studies of binding affinity using recombinant material suboptimal, however, these materials still represent the best option for many *in vitro* studies. Furthermore, binding affinity measurements have utilized the soluble extracellular FcγR domains due to challenges associated with extracting full-length material from the membrane. Tethering CD16a to the membrane changes the N-glycosylation, likely due to differential localization within the Golgi (88, 109). Unfortunately, the N-glycosylation profile of full-length CD16a (frCD16a) expressed with HEK293 cells revealed an N-glycan profile unlike that found on NK cells (109). N-glycans from frCD16a showed less under-processed oligomannose and hybrid types (27% in frCD16a and 45% in NK cell CD16a) and the complex-type N-glycans were highly branched. Thus, cell-type specific glycosylation accounts for the dissimilar N-glycan profile on CD16a from primary and recombinant sources and impacts binding affinity measurement, as discussed below.

Recombinant FcγRs are valuable to characterize the role of N-glycosylation on IgG binding affinity, despite clear differences in N-glycan processing when compared to endogenous material. One recent study reported a 40-fold increase in affinity toward afucosylated IgG1-Fc (G0 form) when complex type N-glycans on CD16a were replaced with Man_5_ N-glycans (110). This gain revealed that CD16a can bind with an affinity comparable to CD64, the “high affinity FcγR.” A comparable study demonstrated that higher amounts of larger sialylated complex type N-glycans on CD16a expressed in CHO cells correlated with lower affinity for Rituximab (108).

Of five CD16a N-glycans, only two appear essential for high affinity interactions. Mutating the protein to eliminate N-glycan addition with N45Q and N162Q substitutions reduced the affinity for IgG1-Fc (109, 115–117). However, the reported influence of N-glycan composition was primarily driven by the N-glycan at N162: only the N162Q mutation abolished the affinity gain due to Man_5_ N-glycans on CD16a (110). These observations are in agreement with the fact that glycans at N45 and N162 form interactions with the CD16a polypeptide and influence protein structure (118) and glycans at these two sites showed the greatest restriction in N-glycan processing using the HEK293 and CHO systems (119). Thus, cell-type specific CD16a N-glycosylation patterns influence affinity for IgG1 and a range of potential affinities are accessible purely through modifying N-glycan processing.

### N-glycosylation of CD16b

CD16b is a highly similar paralogue of CD16a and only found in humans (97% sequence homology of the extracellular antibody-binding domain). However, two common CD16b alleles encode either four (NA1) or six (NA2) N-glycosylation sites (120, 121) (Figure 2). Considerable site-specific diversity in N-glycan structures was present on sCD16b obtained from 2l of pooled human serum (111). Serum sCD16b is generated by ADAM17 cleavage of cell surface CD16b upon neutrophil activation (48). Thus, sCD16b were likely membrane bound when the N-glycans were being processed. The N-glycans at each site had unique profiles ranging from smaller oligomannose type N-glycans at N45 to large complex type N-glycans with extensive elongation, sialylation, and fucosylation at N38 and N74, unlike sCD16b expressed in recombinant systems (106, 114, 122). Additionally, allele specific (NA1 and NA2) N-glycosylation profile at N162 and N45 of donor matched serum and neutrophil CD16b confirmed the observations of CD16b from pooled serum, revealing moderate variability in the abundance of the most prominent glycoforms (123). The profile of sCD16b from serum was distinct from CD16a expressed by NK cells that displayed a greater level of under-processed N-glycans (109, 111). The presence of oligomannose type N-glycans only at N45 strongly suggests under-processing of N-glycan is restricted to a single site on the protein with as many as six N-glycosylation sites (111).

The stark differences in the glycosylation profile of sCD16b from serum compared to recombinant sCD16b further emphasized the importance of cell type specific N-glycosylation. Glycomics analysis of CD16b from HEK293, NS0 and BHK revealed mainly multiantennary complex type N-glycans with a high degree of sialylation and fucosylation (106, 114, 122). The N-glycosylation profile of recombinant sCD16a and sCD16b are comparable as most of the N-glycosylation sites are shared (124). There was a minimal difference (2-fold increase) in affinity when sCD16b-Man5 binding to IgG1-Fc (G0F form) was compared to sCD16b with complex-type N-glycans (110). This was surprising considering that the extracellular antibody binding domains of CD16a and CD16b (NA2) differ at only four amino acid residues. Moreover, both CD16s are functionally distinct because CD16a-complex type has a 15-fold greater affinity for IgG-Fc than CD16b-complex type (110). The affinity and sensitivity to glycan composition for CD16b was improved to that of CD16a by mutating a single residue, Asp129, to Gly based on the CD16a sequence (124). The authors demonstrate with x-ray crystallography and molecular dynamics simulations that Asp129 buckles the CD16b backbone upon binding IgG1 Fc. Thus, buckling shifts a nearby residue, Arg155, which makes a different contact with the N162-glycan that is not observed in CD16a.

### N-glycosylation of CD32

The N-glycosylation profiles of sCD32a and sCD32b expressed with recombinant systems were highly comparable (106, 108, 114). There are two to three N-glycosylation sites on CD32: CD32a (3), CD32b (2), and CD32c (2; 32b and 32c have identical extracellular domains) (Figure 2). Glycomics analysis of CD32a and CD32b expressed in HEK293, NS0, and CHO displayed predominantly biantennary and triantennary complex type N-glycan structures with a low degree of sialylation and varying levels of fucose (106, 108, 110, 114). Binding affinity between sCD32a and sCD32b was comparable and neither appeared sensitive to N-glycan composition as sCD32(a or b)-Man5 and sCD32(a or b)-complex type bound IgG1 Fc with similar affinities (106, 110). CD32a polymorphisms (R131 or H131) cause differences in binding to IgG subtypes, potentially changing the sensitivity of immune complexes to phagocytosis by neutrophils and monocytes (121, 125). However, N-glycan analysis on the receptor expressed in CHO cells showed no substantial difference in glycosylation pattern between the two CD32a allotypes (108). The site-specific N-glycosylation profile and native N-glycosylation profile for any CD32 is not currently available.

### N-glycosylation of CD64 Also Impacts Binding Affinity

The high affinity FcγR, CD64, is distinct from other FcγRs because it contains an additional extracellular domain (126). Moreover, CD64 can potentially receive N-glycosylation modification at seven sites in its extracellular domain (Figure 2). A comparative glycomics analysis of the sCD64 expressed in HEK293, NS0, and CHO cell lines showed biantennary and multi-antennary complex type N-glycans with varying degrees of sialylation and fucosylation as the most abundant glycoforms (106, 108, 114). A distinct feature which was conserved across sCD64 expressed in all three cell lines was the higher abundance of oligomannose structures when compared to recombinant CD16 or CD32. It was speculated that the presence of Man_5_ forms (the most abundant oligomannose N-glycan in these cell types) conferred a stabilizing effect toward IgG1 binding since the higher abundance of Man_5_ forms (14.4% in NS0 and 5.2% in CHO) correlated with an increase in binding affinity to Rituximab (108). According to the authors, the increased affinity was due to the lack of core fucose on the Man_5_ structure which can potentially prevent steric hindrance effects similar to that observed in fucosylated N-glycan on IgG1 (115, 127). The authors also observed that the presence of large sialylated complex type N-glycans on CD64 correlated with reduced binding affinity for Rituximab, indicating that these glycans destabilized the interaction (108). Even though N-glycan composition on CD64 can affect IgG1 affinity, the N-glycosylation profile of native CD64 and the composition of N-glycans at each site remains unknown.

N-glycosylation processing depends on the amino acid sequence and secondary structures which affect the exposure of substrate monosaccharide residues to the glycan processing enzymes (Figure 3). Presence of both the under-processed and highly-processed (tetraantennary sialylated) N-glycan structures on NK cell CD16a and recombinant sCD64 suggests site-specific glycan modification. Oligomannose structures at specific sites on sCD16a have been implicated in modulating IgG affinity; similarly, specific sites on CD64 can be involved in modulating CD64-IgG1 affinity (108, 110). Thus, a thorough analysis of site-specific N-glycosylation analysis of recombinant and endogenous FcγRs from all expressing tissues is required to fully elucidate the role of N-glycosylation pattern at specific sites in affinity modulation.

## How Multivalency Impacts IgG-FcγR Interactions

Investigating factors that contribute to the monovalent affinity of IgG-FcγRs interaction revealed clear differences in the affinity of antibody subclasses for certain receptors, however, multivalent avidity likely determines the *in vivo* immunological response initiated by these interactions. High IgG concentrations in the serum of ~10 mg/ml provide monomeric antibody to the receptors at a concentration of ~67 μM, vastly exceeding the *K*_D_ of IgG1 for all human receptors (7). Thus, surface-borne FcγRs are occupied on cells circulating in the peripheral compartment and multivalent interactions must compete with monomeric IgG to cluster receptors (7, 8). Receptor cross-linking and clustering on the effector cell surface is essential for signal transduction through FcγRs, thus multivalent immune complexes or opsonized targets are the functionally appropriate ligands for the receptors (Figure 1) (54, 128). Distinct FcγRs are engaged depending on the responding cell type, the IgG subclass, the antibody concentration on the opsonized target, and the size of immune complex (Figures 1, 2) (129, 130). Furthermore, the differential binding of immune complexes has therapeutic as well as pathogenic properties, especially during infection and autoimmune disease but not all aspects are well-defined (9, 15). Therefore, defining the critical factors associated with immune complex recognition is required to fully understand the antibody-mediated immune response.

### Immune Complex Size Determines Effector Function

The importance of interactions between multiple monovalent ligands and multiple receptors is well known, however, the study of multivalent interactions remains challenging. Early attempts to generate multivalent immune complexes through heat aggregation of IgG produced aggregates with varied valency, immunogenicity and ill-defined sizes (131, 132). Technological advances in recent years produced immune complexes of defined size and valency which accurately represent those generated *in vivo* (130). Functional interrogation using defined immune complex revealed that immune complex size contributes to interactions with FcγRs.

#### Immune Complex Size Affects Binding

The concentration of antigen-specific antibody in the serum and likewise immune complex size is expected to change during an immune response, and size-associated changes in the immune response are well described (130, 133). Nimmerjahn and coworkers used well-defined immune complexes formed by all four IgG subclasses binding to FcγRs expressed on a CHO cell surface to systematically determine that there was a clear size-dependent gain in binding by IgG2 and IgG4 immune complexes and the size of an immune complex can overcome IgG glycan truncation, a modification that destroys the monovalent interaction (134). Moreover, the binding patterns were comparable to experiments using primary leukocytes that increased cytokine secretion in response to larger immune complexes. These data led to a mathematical model that describes effects of valency and IgG subclass on *in vivo* function (135). The differential binding due to a change in the size of immune complex can potentially lead to substantial changes in cell signaling and recent technical advances provide a means to quantitate signaling with cell-based assays (136).

#### Role of Immune Complex Size in Autoimmune Disorders

The formation of immune complexes with soluble self-antigen is implicated in the pathophysiology of several autoimmune diseases (137). IVIG is a frequent treatment for a variety of autoimmune disorders, but the exact mechanism of action is not known (138). Even though there is a well-documented role of CD32b in decreasing an immune response triggered by autoantibody immune complexes in murine model of immune thrombocytopenia (ITP) (139), a recent study demonstrated that engaging the inhibitory CD32b alone is not responsible for the decrease in phagocytosis of RBC opsonized by autoantibody in human ITP patients. Instead, the direct engagement of IgG by CD64 and CD32a caused the decrease in phagocytosis (140). Surprisingly, though IVIG dimers and multimers are not necessary for therapeutic efficacy in murine models for ITP, small IVIG oligomers provided more potent inhibition of phagocytosis, indicating a role of IVIG immune complexes in blocking pathogenic immune complexes from binding to activating FcγRs (141). Consistent with this observation, immune complexes formed with the anti-citrullinated protein antibodies isolated from rheumatoid arthritis patients bound preferentially to activating and not inhibiting FcγRs expressed on CHO cells (142). Moreover, CD64 on activated neutrophils and CD32a on macrophages were recognized as receptors for the autoantibody immune complex, eliciting the secretion of pro-inflammatory cytokines. These observations formed the basis for developing engineered multivalent immune complexes as therapeutic options.

#### Considerations Regarding Immune Complex Size in Therapeutic Development

Multivalent synthetic immune complexes show promise and may prove useful in the clinic. For example, a trivalent IgG-Fc construct inhibited autoantibody-mediated FcγR-dependent cellular responses in primary human cells and autoimmune murine models (143). Likewise, an engineered hexameric-Fc construct bound to primary differentiated human macrophages and triggered internalization, colocalizing with the activating FcγRs and elicited a decrease in the phagocytosis of antiCD20-coated human B cells and platelets in a murine ITP model (144). The hexameric Fc construct did not trigger internalization of CD32b and exhibited a much shorter serum half-life in animal models than IgG1, however, the inhibition was effective for several days after the initial injection, suggesting a potential for clinical use. In contrast to the approach of preventing the internalization of pathogenic immune complex to block phagocytosis of healthy cells or activating a pro-inflammatory response, a designed bispecific antibody formed larger complexes that neutralized soluble antigens, leading to rapid clearance from serum of a murine model (145). Thus, studies of multivalent IgG-FcγR interactions provide guidance for the development of effective therapeutic options. However, there are multiple antibody and antigen associated factors which govern the antigenicity of immune complexes that must be considered when designing antibodies with defined FcγR-dependent functions.

### Features of the Antibody and Antigen That Impact Antigenicity of the Immune Complex *in vivo*

#### The Ratio of Antibody to Antigen

Antibody concentration relative to antigen changes throughout the progression of an immune response against an infectious pathogen. Considering influenza infection as an example, the B-cell response can take up to 7–14 days to produce antibodies (146). Generally, the antigen-specific antibody titers increased by up to 10.2-fold, depending on the patient, vastly changing the antibody to antigen ratio and the antibody production can be sustained or subside depending on clearance of the organism.

A minimal threshold of antibody density must be surpassed to elicit an immune response during encounters between an opsonized target and effector cell, typically seen during pathogenic infection (147, 148). Antibody concentrations that exceed the threshold lead to an increase in phagocytic activity, as demonstrated by primary mouse bone marrow derived macrophages phagocytosing opsonized sheep erythrocytes. Moreover, at relatively high concentrations of IgG, a valency dependent induction of IL-10 production was seen (148). Similarly, infection with *Cryptococcus neoformans* in mice could be cleared using a specific ratio of antibody to antigen, ratios with excessive antibody led to a detrimental host response mainly due to a reduction in pro-inflammatory cytokines secretion in organs associated with the infection (149). Apart from changes in cytokine secretion potential, larger immune complexes formed with high concentrations of neutralizing antibody against dengue virus actually inhibited antibody-dependent enhancement by binding to the inhibitory receptor CD32b on phagocytic monocytes (150). Thus, relative antibody concentration can modulate immune response in an FcγR-dependent manner by altering the size and concentration of immune complexes; this effect may be similar to the therapeutic benefit of IVIG in autoimmune conditions.

#### Concentration of the Immune Complex

Immune complex concentration likewise impacts viral infection. Apart from the traditional view of Fab-mediated neutralizing activity, Fc dependent effector functions are becoming increasingly recognized in protection against viral infection (16, 17, 151). Classical FcγR-dependent protective mechanisms such as ADCC and ADCP, as well as antibody dependent enhancement of infection, are influenced by the size of the immune complex and IgG subtype coating the viral particle (17, 152). The production of a high concentration of immune complexes are common during chronic viral infection in mice (153). However, high concentrations do not always lead to favorable outcomes. A high concentration of immune complex blocked FcγRs on primary murine macrophages and dendritic cells, negatively impacting viral clearance, and other FcγR-related activity (153). These phenomena were independent of CD32b and reversed once the immune complex concentration was reduced. Thus, the role of FcγRs during pathogen infection is complex and varied but there is a clear dependence of cellular response based on immune complex size and concentration, similar to that observed in autoimmune disease discussed above.

#### Affinity of the Antibody for Antigen

At a fixed antibody concentration, the affinity of the antibody toward the antigen can determine how many Fcs are displayed on the immune complex and are available to interact with FcγRs (154). A recent study showed that at saturating concentrations, antibodies with high affinity for antigen elicited a weaker ADCC response compared to antibodies with lower affinity (*K*_D_ = 0.8 nM and 72 nM, respectively) (155). The observed difference in the immune response was attributed to the higher proportion of monovalent antigen binding displayed by the lower affinity antibody, recruiting a larger number of antibodies to the cell surface and increasing the number of Fcs available to the leukocyte. A notable feature of this observation is the initial IgG response often produces antibodies with antigen-binding affinities similar to the lower affinity antibody in this study. Antibody concentration and antibody-antigen affinity are not the only factors affecting immunogenicity of immune complex. A comparative analysis of three anti-TNFα antibodies with a range of affinities (*K*_D_ = 0.18–5.1 nM) showed that the size and composition of the immune complex was determined by the properties associated with epitope location and binding energetics (156).

#### Epitope and Antigen Location

Location of the epitope influences the immune response. Neutralizing antibodies targeting the stalk region of the influenza hemagglutinin protein induced FcγR-dependent cytotoxicity while antibodies binding the head domain did not (12). A comparable analysis of anti-Ebola antibodies showed that binding to the most membrane distal portion of viral surface glycoprotein elicited the highest ADCP and antibody-dependent neutrophil phagocytosis (ADNP) compared to antibodies that bound to the membrane proximal regions (157). Even though epitope location on the antigen is not directly implicated in changes in immune complex size in these studies, it is likely that the epitope location causes changes in immune complex properties since three different monoclonal antibodies against different epitopes on sCD154 and TNFα also formed different immune complexes (156, 158). In other cases, the height of the antigen from the target surface affected phagocytosis in a valency-independent manner (55). Antigens which are < 10 nm from target surface promoted phagocytosis when compared to antigens further away from the surface because close contact between target and effector cell surface was necessary to exclude effector cell the inhibitory CD45 from the immune synapse following FcγRs clustering (as noted above). Additionally, antibodies binding West Nile virus epitopes that are normally buried can form immune complexes, given sufficient incubation time, though these immune complexes are smaller and led to lower neutralization levels (154). Thus, location of the epitope can affect the immune response but the effect of epitope location on immune complex size is not fully understood.

The location of the antigen (soluble or cell bound) affects FcγR clustering and the subsequent immune response. A soluble antigen may form relatively smaller immune complexes which are endocytosed but a cell surface antigen forms a relatively larger opsonized target that is more likely phagocytized as determined using mouse bone marrow-derived macrophages (133). Both mechanisms, triggered through FcγRs, are distinct and induce different signaling and subsequent immune responses (128, 159). In one example, small soluble immune made with soluble CD154 would be expected to be endocytosed, and CD154 tethered to a T cell membrane led to the formation of very large complexes at the cell surface (158). Surprisingly, the specific monoclonal antibody greatly influenced the immune complex structure. It is also known that opsonized targets can exhibit lateral diffusion on the leukocyte surface which also affects the multivalent interaction with FcγRs (160).

#### Malleable vs. Rigid Target Surfaces

In addition to size and shape, deformability of the target also impacts activation. The phagocytosis of opsonized polyacrylamide beads tuned to exhibit different rigidity established that phagocytosis of ridged particles was preferred over relatively more deformable particles by mouse bone marrow-derived macrophages (161). A related study demonstrated that murine macrophage RAW264.7 cells phagocytosed emulsion droplets at a lower IgG concentration when compared to solid particles (162). It was speculated that the attachment of IgG on the surface of rigid particles prevents the lateral diffusion of opsonizing antibodies, while lateral diffusion was observed in opsonized emulsion droplet. Thus, the location of the antigen, which facilitated higher cell surface FcγRs interaction at lower antibody concentrations, can affect recognition of the complex.

#### IgG-Subclass Impact Immune Response

FcγR binding is also affected by IgG subclass. Specificity of a specific IgG subclass binding to a FcγR is largely studied in context of a monovalent interaction (23), however, immune complexes and opsonized target cells are the natural ligands. Additionally, specific IgG subclasses are related to various disorders indicating immune complex composition is important (1, 152, 163). Therefore, studying these interactions in a multivalent form is required to accurately determine their binding properties and the subsequent immune response. The observation that immune complexes of certain IgG subclasses only bind at higher concentrations indicates that IgG subclass is also a variable which can affect the immune response (164).

The Fcs of different IgG subclasses have distinct amino acid residues and hinge regions which can affect binding to the FcγRs, despite a high degree of sequence conservation (Figure 2) (1). A systematic analysis of multivalent binding for the four human IgG subclasses to the cell surface FcγRs revealed the IgG2 and IgG4 subclasses, which showed minimal affinity in a monovalent interaction, bound as immune complexes to FcγRs expressed on CHO cells at higher concentrations (164). This study also demonstrated that allotype variants of FcγRs had different binding properties toward immune complexes generated by different IgG subtypes. CD16a V158 bound IgG3 immune complexes with high affinity while CD16a F158 bound more weakly and CD32a H131 had a higher affinity to IgG2 immune complex compared to CD32a R131. Another report showed that the CD32a H131 variant bound to IgG1, IgG2, and IgG3 with higher affinity than CD32a R131. This observation may explain why the CD32a R131 allotype is associated with greater susceptibility to bacterial infections and autoimmune disorders (163). Thus, the wide range of binding affinities displayed by FcγRs toward IgG subclass specific immune complexes can impact clinical outcome.

The use of different IgG subclasses in designed immune complexes can also impact potential therapeutic use. Incubation of a hexameric IgG1 Fc construct, discussed above as an inhibitor of phagocytosis, elicited the release of higher cytokine levels in whole blood when compared to PBMCs, likely due to CD16b engagement on neutrophils (not present in PBMCs) (165). Furthermore, the hexameric IgG1 Fc construct also triggered release of cytokines from platelets through a CD32a-dependent interaction. However, a hexameric IgG4 Fc construct did not promote the release of cytokines from neutrophils or platelets. This result is consistent with the reduced affinity of IgG4 for CD16b and CD32a when compared with IgG1, highlighting the potential utility of specific FcγR interactions.

## Summary

The multitude of factors influencing the immune system each affects a wide range of responses. This review covers a relatively limited collection of variables that contribute to an FcγR-dependent immune response (Figure 1). There appear to be few inviolable laws governing this aspect of the immune system, and every newly discovered variable introduce a new handle to tune the immune response, at least *in vitro*. It is well known that different monoclonal antibodies to a single target elicit different responses, in many cases through the mechanisms described here. If any lessons are to be learned, it is that each antibody must be thoroughly evaluated using systems that recapitulate as closely as possible endogenous immune system components. One striking example of this tenet is the observation that the efficacy of a hexameric IgG1 Fc increased when neutrophils and platelets were incorporated in an *in-vitro* assay with PBMCs (165). Moreover, soluble complement components can also bind the immune complex to affect the immune response as reported in few studies described above (130, 148, 149). Laboratory studies often focus on immune complexes formed by monoclonal antibodies, but that is likely not the case *in vivo* with a polyclonal immune response to vaccines or infection; one study demonstrated that a mixture of disease neutralizing and disease enhancing antibodies against *Bacillus anthracis* formed immune complexes that elicited a protective immune response (166). Thus, these observations highlight the complex yet important features associated with studying FcγRs function *in vivo*.

Animal models have, and will continue to have, an important role in studies designed to understand human FcγRs in immune function. Despite the differences in FcγR cellular expression patterns and minor differences in binding affinities to human IgG subclass, animal (mainly murine and non-human primate) models have sufficiently recapitulated human FcγR biology to be used for studying FcγR function and test therapeutic molecules (167–173). A recent study determined that the mouse FcγRIV and the human equivalent to human CD16a both share the conserved N-glycosylation site at N162 which mediates tight binding to afucosylated mouse IgG similar to observations in human system, and human IgG binds mouse FcγRs with similar affinity patterns as human FcγRs demonstrating conservation of certain functional features of human FcγR biology in mouse model (170, 174). Furthermore, several studies mentioned in this review have employed murine autoimmune models, humanized models, cell lines or primary cells to test efficacy of engineered antibody products and delineate mechanistic aspects of the FcγRs dependent cellular response, demonstrating that these models are indispensable for understanding human FcγR biology (61, 66, 139, 141, 143, 162). The two successful strategies to attain humanized FcγR mouse models eliminate the influence of mouse FcγRs in studying human FcγR function in these models and can uncover novel role of FcγRs in autoimmune disorders, infection and cancer immunity (175, 176). However, important yet undefined FcγR variables including post-translational modification including glycosylation as well as copy number variation and interaction with coexpressed membrane proteins likely vary in animal models. It is likely that organism diversity in these key variables likewise differentially impacts immune function, comparable to the diversity attributed to protein coding regions and gene variability between species.

It is worth highlighting the role of post translation modification of the FcγRs as another critical variable that is overlooked due to the historical inability to resolve differences in the glycosylation of endogenous material. One future challenge will be matching the level of detail known regarding serum IgG glycosylation with studies of functionally-relevant FcγR modifications as these have the potential to exert an enormous influence on the immune response. Differential gene expression profiles of the glycan modifying enzymes are present in monocytes, dendritic cells, and macrophages, suggesting the potential for the functionally-relevant differentiation and maturation specific N-glycosylation modifications (177). A complete understanding of the immune response will require the definition of these recently discovered variables, with the likelihood that more variables will emerge.

## Author Contributions

All authors listed have made a substantial, direct and intellectual contribution to the work, and approved it for publication.

### Conflict of Interest Statement

The authors declare that the research was conducted in the absence of any commercial or financial relationships that could be construed as a potential conflict of interest.
